# Predominantly myalgic phenotype caused by the c.3466G>A p.A1156T mutation in *SCN4A* gene

**DOI:** 10.1212/WNL.0000000000003846

**Published:** 2017-04-18

**Authors:** Johanna Palmio, Satu Sandell, Michael G. Hanna, Roope Männikkö, Sini Penttilä, Bjarne Udd

**Affiliations:** From the Neuromuscular Research Center (J.P., S.P., B.U.), Department of Neurology, Tampere University and University Hospital, Neurology; Seinäjoki Central Hospital (S.S.), Department of Neurology, Finland; MRC Centre for Neuromuscular Disease (M.G.H., R.M.), UCL Institute of Neurology, Queen Square, London, UK; Folkhälsan Institute of Genetics and the Department of Medical Genetics (B.U.), Haartman Institute, University of Helsinki; and Vaasa Central Hospital (B.U.), Department of Neurology, Finland.

## Abstract

**Objective:**

To characterize the clinical phenotype in patients with p.A1156T sodium channel mutation.

**Methods:**

Twenty-nine Finnish patients identified with the c.3466G>A p.A1156T mutation in the *SCN4A* gene were extensively examined. In a subsequent study, 63 patients with similar myalgic phenotype and with negative results in myotonic dystrophy type 2 genetic screening (DM2-neg group) and 93 patients diagnosed with fibromyalgia were screened for the mutation. Functional consequences of the p.A1156T mutation were studied in HEK293 cells with whole-cell patch clamp.

**Results:**

The main clinical manifestation in p.A1156T patients was not myotonia or periodic paralysis but exercise- and cold-induced muscle cramps, muscle stiffness, and myalgia. EMG myotonic discharges were detected in most but not all. Electrophysiologic compound muscle action potentials exercise test showed variable results. The p.A1156T mutation was identified in one patient in the DM2-neg group but not in the fibromyalgia group, making a total of 30 patients so far identified. Functional studies of the p.A1156T mutation showed mild attenuation of channel fast inactivation.

**Conclusions:**

The unspecific symptoms of myalgia stiffness and exercise intolerance without clinical myotonia or periodic paralysis in p.A1156T patients make the diagnosis challenging. The symptoms of milder *SCN4A* mutations may be confused with other similar myalgic syndromes, including fibromyalgia and myotonic dystrophy type 2.

Mutations in the sodium channel gene *SCN4A* encoding the Nav1.4 voltage-gated sodium channel are well-known causes of the skeletal muscle channelopathies: paramyotonia congenita (PMC), other forms of myotonia, and periodic paralyses (hyperkalemic periodic paralysis [HyperPP], normokalemic, and hypokalemic periodic paralysis). To date, >40 dominant mutations in *SCN4A* have been reported to cause variable phenotypes, depending on the type and location of the mutations.^1,2^ In addition, rare recessive *SCN4A* mutations have been associated with congenital myasthenia or congenital myopathy.^3,4^ The type of channel defect and the degree of depolarization account for clinical symptoms; membrane hyperexcitability causes myotonia, increased membrane depolarization, and inexcitability, leading to paralysis,^5–7^ while membrane hypoexcitability underlies myasthenias and myopathies.

The c.3466G>A p.A1156T mutation in the *SCN4A* gene was originally reported in a family of Finnish origin with incomplete penetrance. The phenotype varied and consisted of features of HyperPP, PMC, and myotonia.^8^ We now report clinical and electrophysiologic findings in 30 Finnish patients with the c.3466G>A p.A1156T mutation, including 1 patient found in the screening study. Their unspecific, predominantly myalgic phenotype was very consistent in all patients lacking most of the other typical features of *SCN4A* channelopathies. The relatively modest gain in the function of p.A1156T channel may explain this unusual clinical phenotype often lacking myotonic findings.

## METHODS

### p.A1156T patients.

**Figure 1:**
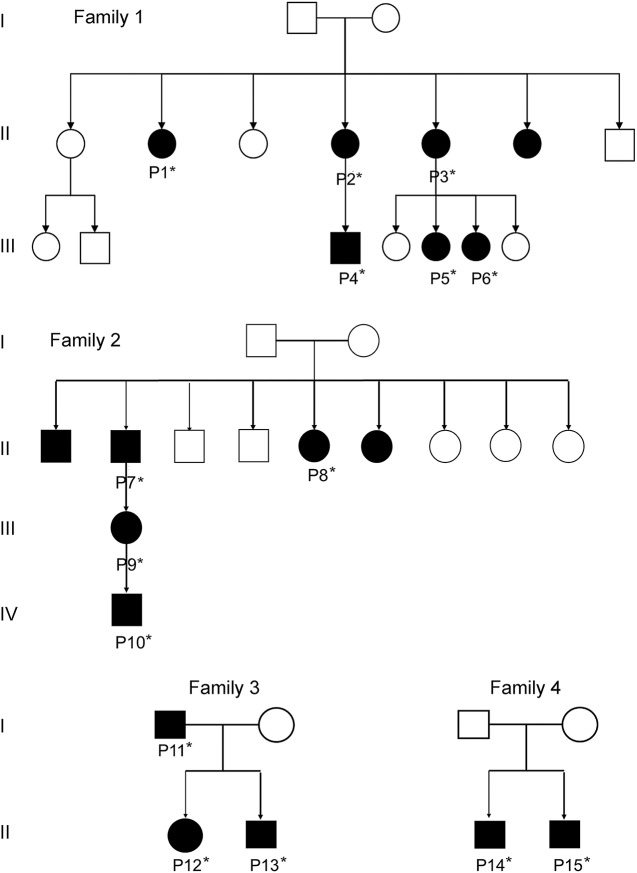
Pedigree of the families *DNA available.

We identified 29 Finnish patients from 18 different families who had similar clinical phenotype and a confirmed c.3466G>A p.A1156T mutation in *SCN4A* (table e-1 at Neurology.org). The index family (F1) consisted of 7 affected members who had muscle stiffness and myalgia (figure 1). Three of them had an earlier diagnosis of congenital myotonia based on myotonic discharges observed on EMG examination, but when genetic testing became available, the genetic defects in the chloride channel gene *CLCN1* could be ruled out. Four families (F1–F4) had several affected members in 2 or 3 generations (figure 1); others were single patients, but many of them had family members with similar muscle symptoms. The patients underwent clinical examinations by neurologists, including manual muscle strength evaluation, assessment of clinical myotonia (grip myotonia, percussion myotonia, and eyelid myotonia) and paramyotonia (repeated action myotonia), creatine kinase (CK) levels, and electrophysiologic studies. Muscle biopsy was available in 12 patients. Three patients were also examined by muscle MRI.

### Screening study of myotonic dystrophy type 2–negative and fibromyalgia patients.

Sixty-three patients who were earlier suspected to have myotonic dystrophy type 2 (DM2) on the basis of exercise-induced myalgia, stiffness, and EMG findings and who had had negative results in the genetic testing for the *CNBP* repeat expansion mutation causing DM2 were included in the screening study (DM2-neg group). They had normal or slightly elevated CK levels (up to 2 times the upper normal limit), no evidence of fixed muscle weakness, and minor findings on muscle biopsy, i.e., small 2A fibers or increased amount of internal nuclei. On EMG, they had either increased insertional activity or some myotonic discharges. In addition, 93 patients with a diagnosis of fibromyalgia but no suspicion of myopathy were screened for *SCN4A* exon 19 mutations.

### Electrophysiologic studies.

Standard neurography and EMG investigation was performed in 25 patients with p.A1156T. At minimum, 2 motor and 2 sensory nerve conduction studies were performed, and both proximal and distal muscle groups investigated from at least 1 upper and 1 lower limb. Compound muscle action potentials (CMAP) exercise test was carried out in 14 patients. A Fournier protocol was used^9,10^ consisting of short (10–12 seconds) and long (5 minutes) exercise tests. CMAP were evoked by supramaximal nerve stimulation. Tested muscles were the abductor digiti minimi and extensor digitorum brevis.

### Muscle biopsy.

Routine diagnostic histologic and histochemical stainings were performed in 12 patients. In addition, chloride channel CLC-1 immunohistochemistry was performed in 4 samples with a published method.^11^

### Genetic studies.

Genomic DNA was extracted from leukocytes by standard methods. At the beginning of the investigations, 13 patients were tested for congenital myotonia (*CLCN1*, at least the 3 most common mutations in Finland), 14 for DM2 (*CNBP* repeat expansion mutation), and 2 for DM1 (*DMPK* repeat expansion mutation). There was a heterozygous known recessive mutation in *CLCN1* gene in 2 patients, whereas other results were negative. For all patients but one (P13), the whole coding sequence of exon 19 of *SCN4A* was screened. The region studied was amplified by PCR and directly sequenced with the Big-Dye Terminator version 3.1 kit on an ABI3130xl automatic DNA sequencer system (Applied Biosystems, Foster City, CA). Sequences were analyzed with Sequencher 5.1 software (Gene Codes Corporation, Ann Arbor, MI). The primers used are available on request. For P13, the genetic analysis was performed using targeted next-generation sequencing as previously described^12^ with version 2 of the MYOcap gene panel that is targeted to the exons of 236 genes known or predicted to cause muscular dystrophy or myopathy.

### Mutagenesis, in vitro transcription, HEK293 cell transfection, and whole-cell patch clamp.

The human *SCN4A* expression clone was a gift from S.C. Cannon (University of Texas Southwestern Medical Center, Dallas). Site-directed mutagenesis was performed with the QuikChange kit (Stratagene) and confirmed by sequencing the entire insert. HEK293 cells were transfected with 0.5 μg wild-type or mutant plasmid together with 50 ng plasmid coding for cop-GFP with Lipofectamine 2000 (Invitrogen, Waltham, MA) in a 1.9-cm^2^ well. HEK293 cells with green fluorescence were voltage clamped at room temperature 48 to 72 hours after transfection with Axopatch 200B, Digidata 1440B, and pClamp software (all Axon Instruments, Sunnyvale, CA). Extracellular solution was (in mmol/L): NaCl 145, KCl 4, MgCl2 1, CaCl2 2, and HEPES 10, pH 7.4 (NaOH). Patch electrodes were filled with pipette solution (in mmol/L): NaCl 5, CsCl 145, EGTA 10, and HEPES 10, pH 7.3 (CsOH). Liquid junction potential was estimated at −4.4 mV and not corrected for. Currents were low-pass filtered at 5 kHz and sampled at 50 kHz. Series resistance error was kept below 5 mV. Data were analyzed and illustrated with pClamp, Origin (OriginLab, Northampton, MA), and Excel (Microsoft, Redmond, WA) software. The voltage protocols are described in the figure legends. The current- and conductance-voltage relationships were fitted with the Boltzmann equation: G = A + (B − A)/{1 + exp[(V_1/2_ − V)/V_slope_)]}, where A and B are the maximum and minimum amplitudes, V_1/2_ is the voltage where amplitude is (A − B)/2, and V_slope_ is the slope factor. The time course data of recovery from inactivation and of onset of fast inactivation were fitted with single or double exponential functions, respectively. Statistical comparisons were performed with the Student *t* test.

### Standard protocol approvals, registrations, and patient consents.

The study was approved by the Institutional Review Board of Tampere University Hospital. All participants provided appropriate consent, and the study was conducted according to the Helsinki Declaration.

## RESULTS

### Clinical and genetic findings of the patients with p.A1156T.

In the patient cohort, 17 were female and 12 were male. The age at onset of symptoms ranged from 4 to 53 years (mean 28.7 years). However, only 3 patients reported an early-childhood onset of muscle symptoms; the more typical age at onset was between 20 and 40 years. The common clinical features were exercise and cold-induced muscle cramps, muscle stiffness, and myalgia. Several patients also experienced muscle weakness or fatigue during and shortly after exercise, although without paralytic episodes (e.g., paralysis at rest after exercise). Muscle strength was normal in all but 2 patients on clinical examination. CK level was slightly increased in 1 patient (2.5 times the upper normal). Muscle biopsies showed mild abnormalities, i.e., rare highly atrophic fibers or increased internal nuclei. However, the findings were considered normal in 7 patients. Clinical myotonia (grip, percussion, eyelid myotonia) or typical paramyotonia (repeated action myotonia) was not evident on clinical examination. The diagnosis of fibromyalgia was very common among the family members of the patients. Clinical details of each patient are shown in table e-1.

All 29 patients were identified with the heterozygous c.3466G>A p.A1156T mutation.^8^ Two unrelated patients, P16 and P18, were also carriers of the recessive c.2680C>T p.R894X mutation in the *CLCN1* gene. Both had normal CLC-1 protein expression as judged by immunohistochemical staining, indicating no additional defect in the corresponding gene.^11^

### Screening of the DM2-neg and fibromyalgia groups.

No mutations were observed in the fibromyalgia group. In the DM2-neg group, one patient (P30) was found to harbor the p.A1156T mutation. She had exercise-induced myalgia and cramps starting at age 28. Clinical examination was normal, and there was no electrophysiologic myotonia, but EMG showed increased insertional activity with uncharacteristic repetitive discharges. She had occasional elevated CK levels (2 times the upper normal limit), and her muscle biopsy from gastrocnemius medialis muscle was normal.

### Muscle MRI.

Two siblings in family 3 had mild degenerative changes in vastus lateralis muscles, although muscle strength was normal on manual testing. One patient (P22) had mild degenerative findings on MRI in the left thigh muscles. She had had a fracture of the left femur 10 years earlier due to fibrous dysplasia of bone that might contribute to the imaging findings and to the clinical finding of muscle weakness on the left lower limb. The one patient from the DM2-neg group who had the p.A1156T mutation in the screening study showed normal muscle MRI findings at the age of 30.

### Electrophysiologic findings.

EMG myotonic discharges were detected in most but not all patients. EMG was not studied in 5 symptomatic mutation carrier family members of the probands. Sixteen patients had myotonia on EMG at some point, but interestingly, in 6 of them, myotonic discharges disappeared on repetitive examinations with more advanced age (table 1). The rest of the patients showed increased insertional activity only. Thus, none of the initial EMG studies were completely normal. Fourteen patients underwent CMAP exercise test (Fournier protocol) with variable results. It was normal in 6 patients; 3 patients showed a 35% to 55% decrease of CMAP amplitude after the short exercise; 4 patients had a 28% to 60% decrease after the long exercise; and 2 patients had a 30% to 44% decrease after cooling.

**Table 1:**
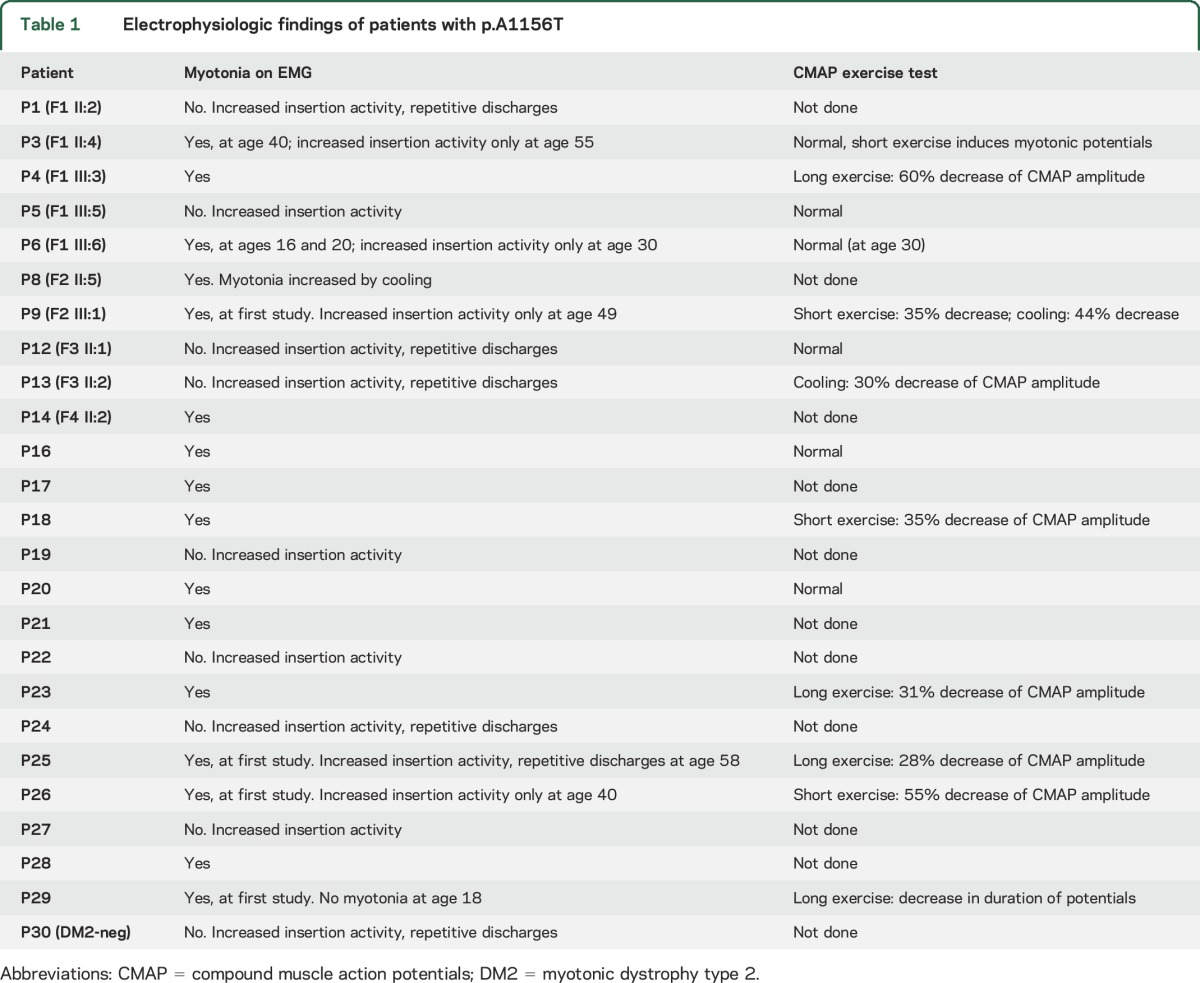
Electrophysiologic findings of patients with p.A1156T

### Functional studies.

**Figure 2:**
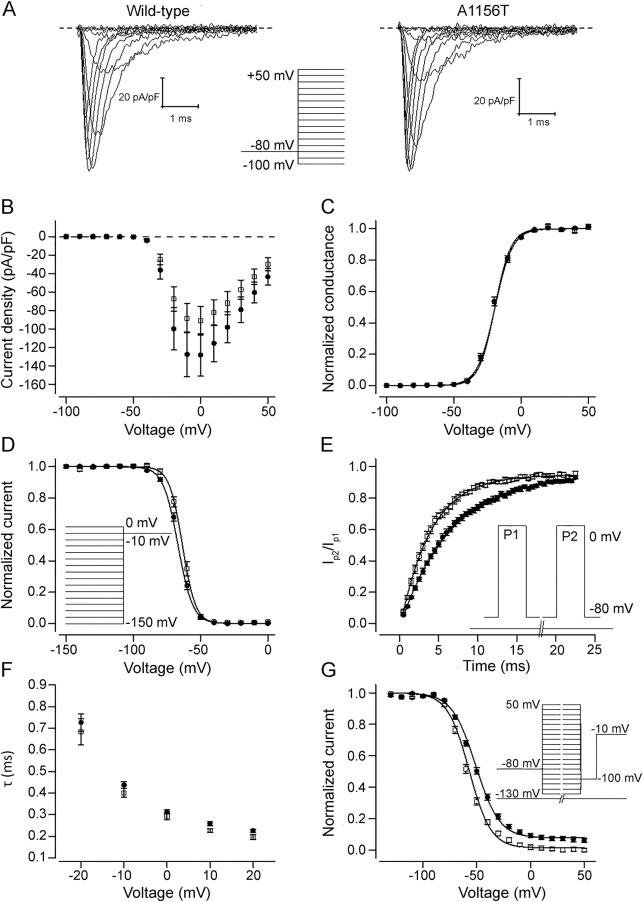
Functional characterization of p.A1156T channel (A) Representative current traces of wild-type and p.A1156T channels in response to test voltages ranging from −60 to 50 mV. Scale bars are 1 millisecond (x-axis) and 20 pA/pF (y-axis). Dashed lines indicate 0 current level. Voltage protocol for voltage steps ranging from −150 to 50 mV is shown in inset. (B) Peak current amplitude in response to test voltages ranging from −150 to 50 mV in 10-mV increments is plotted against the test voltage for wild-type (solid circles) (n = 18) and p.A1156T (open squares) (n = 11) channels. (C) Voltage dependence of activation was estimated by plotting the conductance [peak current/(test voltage − reversal voltage)] against the test voltage for wild-type (solid circles) (V_1/2_ = −20.3 ± 0.7 mV, n = 18) and p.A1156T (open squares) (V_1/2_ = −20.4 ± 0.7 mV, n = 11) channels. Individual data were normalized to maximum and minimum amplitude of the Boltzmann fit and averaged. Solid lines represent the fit of the Boltzmann equation to the mean data. (D) Voltage dependence of fast inactivation was estimated by plotting the peak tail current amplitude at −10 mV after 150-millisecond prepulse voltage steps ranging from −150 to 0 mV in 10-mV increments against the prepulse voltage for wild type (solid circles) (V_1/2_ = −66.2 ± 0.7 mV, n = 18) and p.A1156T (open squares) (V_1/2_ = −63.5 ± 1.0 mV, n = 11) channels. The voltage protocol is shown on the right. Individual data were normalized to maximum and minimum values of the Boltzmann equation and averaged. Solid lines represent the fit of the Boltzmann equation to the mean data. (E) To estimate the time course of recovery from the inactivation, the current in response to a second voltage pulse to 0 mV (P2) divided by the current in response to the first 10-millisecond pulse to 0 mV (P1) is plotted against the duration of the recovery step at −80 mV between the 2 pulses. Data are shown for wild-type (solid circles) (τ = 6.5 ± 0.4 milliseconds, n = 16) and p.A1156T (open squares) (τ = 3.8 ± 0.3 milliseconds, n = 10) channels. The voltage protocol is shown in the inset. The solid lines represent the fit of exponential function to the mean data. (F) Time course of open-state inactivation was estimated by fitting a double exponential function to the current decay between 90% of peak current amplitude and the current baseline. Time constant (τ) of inactivation at voltages ranging from −20 to 20 mV is shown for wild type (solid circles) (n = 18) and p.A1156T (open squares) (n = 11) channels. Voltage protocol was as in panel (A). Only the time constant of the fast component that carries ≈95% of the amplitude of the inactivating current is shown. (G) Voltage dependence of slow inactivation was studied by plotting the peak tail current amplitude at −10 mV after a 10-second prepulse to voltage steps ranging from −130 to 50 mV in 10-mV increments against the prepulse voltage for wild-type (solid circles) (V_1/2_ = −51.2 ± 0.9 mV, n = 12) and p.A1156T (open squares) (V_1/2_ = −58.3 ± 1.0 mV, n = 7) channels. Between the prepulse and the test pulse, the voltage was stepped to −100 mV for 20 milliseconds to allow the channel to recover from fast inactivation. The voltage protocol is shown in the inset. Individual data were normalized by dividing with maximum value of the Boltzmann equation and averaged. Solid lines represent the fit of the Boltzmann equation to the mean data.

When expressed in HEK293 cells, the p.A1156T channels activated normally (figure 2, A–C). Fast inactivation of p.A1156T channel was mildly attenuated compared to the wild-type channel: V1/2 was shifted <3 mV to depolarized voltages (*p* < 0.05) (figure 2D), and the recovery from inactivation was accelerated 1.7-fold (*p* < 0.001) (figure 2E). No significant changes in the rate (figure 2F) or completeness of the open-state inactivation were detected (not shown). The voltage dependence of slow inactivation of p.A1156T channel was shifted 7 mV to the hyperpolarized direction compared to the wild-type channel (figure 2G) (*p* < 0.001).

## DISCUSSION

Our larger cohort of 30 patients carrying the c.3466G>A p.A1156T mutation in the *SCN4A* gene showed a consistent phenotype of predominant myalgia, muscle stiffness, and exercise cramps without signs of clinical myotonia, paramyotonia, or periodic paralyses. This also explains the incomplete penetrance of myotonia reported in the original family.^8^ Predominant myalgic phenotype expands the spectrum of *SCN4A* channelopathies and interferes with the very large group of patients with myalgia. Relatively modest gain in the function of p.A1156T channel in whole-cell patch clamp studies may explain the absence of clinical myotonia.

Mutations in *SCN4A* gene typically produce several different subtypes of skeletal muscle disorders consisting mainly of clinical and electrophysiologic myotonia or periodic paralyses. Different mutations usually account for different ion channel characteristics and phenotypes, although some of the mutations can cause several phenotypes.^2^ The c.3466G>A p.A1156T mutation has been reported with most of the *SCN4A* manifestations: PMC, HyperPP, and pure myotonia.^8,13^ The articles describing these patients did not report myalgia as part of the clinical manifestation. The main symptom in the first reported family with the p.A1156T mutation was HyperPP, which none of our patients demonstrated.^8^ Of 2 unrelated Korean patients identified with c.3466G>A p.A1156T, 1 patients had myotonia with a warm-up phenomenon resembling chloride channel myotonia, and the other had HyperPP. Myotonic discharges were present on EMG in both patients.^13^ The clinical presentation in our larger patient series was not consistent with periodic paralysis or paramyotonia, although many patients had experienced worsening of the symptoms in cold and some during repetitive exercise resembling paradoxical myotonia. Two patients with an additional heterozygous *CLCN1* mutation did not differ largely from the rest of the patients, although heterozygous *CLCN1* mutations have been found to modulate phenotype in sodium channel myotonia.^14^

The findings in electrophysiologic studies, both regular and CMAP exercise tests, were variable and did not directly show typical sodium channel patterns, nor were they clearly compatible with clinical symptoms as previously suggested.^9,10,15^ Although most of the studied patients had myotonia on EMG at some point, they did not show clinical myotonia, and furthermore, myotonic discharges could disappear later in life, as was observed in 6 of our patients. The findings on the CMAP exercise test were normal in 43% of the patients studied and inconsistent in the rest.

Our functional data are consistent with previous reports showing attenuated fast inactivation of p.A1156T channels.^16–18^ The p.A1156T channels showed a mild shift in the voltage dependence of fast inactivation and acceleration of recovery from fast inactivation. However, in contrast to the previous reports, we did not find defects in the time course of open-state fast inactivation or signs of persistent late currents. While the functional expression reports on A1156T show some discrepancies, defective fast inactivation, in particular accelerated recovery from inactivation, is a consistent feature.^16–18^ The gain of function caused by attenuated inactivation is consistent with myotonic phenotype. The relatively modest shift in the voltage dependence of fast inactivation and acceleration in the rate of recovery from inactivation and the absence of defects in the open-state inactivation may underlie the milder clinical phenotype in which clinical myotonia is not the predominant presentation. In addition, it has been suggested that attenuated slow inactivation is common for *SCN4A* variants associated with periodic paralysis. The slow inactivation of p.A1156T channel was enhanced compared to the wild-type channel, consistent with the absence of periodic paralysis in our p.A1156T cohort.^18^

Myalgia is a very common symptom in the general population, but it is only rarely caused by an underlying muscle disease. At present, no useful guidelines exist on how to clarify the cause of myalgia.^19^ Painful cramps or myalgia with myotonia have been reported in a few families with other *SCN4A* mutations,^20–23^ but to the best of our knowledge, myalgia without myotonic discharges on EMG has not been reported before. Besides p.A1156T *SCN4A* mutation, DM2 is an important disorder to be considered in differential diagnostics of myalgic syndromes. In the early stages of DM2, the symptoms resemble those of p.A1156T because clinical or EMG myotonia also can be absent in DM2.^24,25^ The age at onset of muscle symptoms was earlier in our cohort than in typical cases of DM2. Clearly, elevated CK levels, fixed muscle weakness, or other myopathic signs on muscle histology, imaging, or EMG suggest DM2 rather than milder form of *SCN4A* disease. EMG seems to be a useful tool because the findings were not completely normal in any of our patients. However, the mildest change, i.e., increased insertional activity, can be easily overlooked and not considered clinically relevant.

In the Exome Aggregation Consortium database, the mutation c.3466G>A p.A1156T has been identified in 2 of 3,307 Finnish individuals. If this allele frequency of the mutation is representative for the whole population, there are some 3,000 individuals carrying the mutation in Finland. Even with a lower frequency and considering the usual adult onset of symptoms, it can be estimated that some 1,000 patients in Finland may have myalgia, stiffness, and cramps caused by this mutation.

Milder mutations in *SCN4A* gene may thus underlie myalgic syndromes, especially if the patient has cold-induced worsening of the symptoms and increased insertional activity with or without myotonic discharges on EMG.

## GLOSSARY

CK: creatine kinase
CMAP: compound muscle action potentials
DM2: myotonic dystrophy type 2
HyperPP: hyperkalemic periodic paralysis
PMC: paramyotonia congenita
